# Symptom, diagnosis and mortality among respiratory emergency medical service patients

**DOI:** 10.1371/journal.pone.0213145

**Published:** 2019-02-28

**Authors:** Tim Alex Lindskou, Laura Pilgaard, Morten Breinholt Søvsø, Torben Anders Kløjgård, Thomas Mulvad Larsen, Flemming Bøgh Jensen, Ulla Møller Weinrich, Erika Frischknecht Christensen

**Affiliations:** 1 Centre for Prehospital and Emergency Research, Department of Clinical Medicine, Aalborg University, Aalborg, Denmark; 2 Unit of Business Intelligence, North Denmark Region, Aalborg, Denmark; 3 Emergency Medical Services, North Denmark Region, Aalborg, Denmark; 4 Department of Respiratory Diseases, Aalborg University Hospital, Aalborg, Denmark; 5 The Pulmonary Research Center, Aalborg University Hospital, Aalborg, Denmark; 6 Clinic for Internal and Emergency Medicine, Aalborg University Hospital, Aalborg, Denmark; Azienda Ospedaliero Universitaria Careggi, ITALY

## Abstract

**Objective:**

Breathing difficulties and respiratory diseases have been under-reported in Emergency Medical Services research, despite these conditions being prevalent with substantial mortality. Our aim was two-fold; 1) to investigate the diagnostic pattern and mortality among EMS patients to whom an ambulance was dispatched due to difficulty breathing, and 2) to investigate the initial symptoms and mortality for EMS patients diagnosed with respiratory diseases in hospital.

**Methods:**

Population-based historic cohort study in the North Denmark Region 2012–2015. We included two patient groups; 1) patients calling the emergency number with *breathing difficulty* as main symptom, and 2) patients diagnosed with *respiratory diseases* in hospital following an emergency call. Main outcome was estimated 1- and 30-day mortality rates.

**Results:**

There were 3803 patients with the symptom *breathing difficulty*, nearly half were diagnosed with *respiratory diseases* 47.3%, followed by *circulatory diseases* 13.4%, *and symptoms and signs* 12.0%. The 1-day mortality rate was highest for *circulatory diseases*, then *respiratory diseases* and o*ther factors*. Over-all 30-day mortality was 13.2%, and the highest rate was for *circulatory diseases* (17.7%) then *respiratory diseases* and *other factors*. A total of 4014 patients were diagnosed with *respiratory diseases*, 44.8% had the symptom *breathing difficulty*, 13.4% *unclear problems* and 11.3%. *chest pain/heart disease*. 1-day mortality rates were highest for *decreased consciousness*, then *breathing difficulties* and *unclear problem*. Over-all 30-day mortality rates were 12.5%, the highest with symptoms of *decreased consciousness* (19.1%), then *unclear problem* and *breathing difficulty*. There was an overlap of 1797 patients between the two groups.

**Conclusions:**

The over-all mortality rates alongside the distribution of symptoms and diagnoses, suggest the breathing difficulty patient group is complex and has severe health problems. These findings may be able to raise awareness towards the patient group, and thereby increase focus on diagnostics and treatment to improve the patient outcome.

## Introduction

### Background

Respiratory failure is one of the “First Hour Quintet” (alongside cardiac arrest, myocardial infarction, trauma, and stroke*)* which have been defined as the five time-critical conditions where immediate prehospital care by the Emergency Medical Services (EMS) yield the greatest effect, albeit rarely studied [1,2]. Dyspnoea, or breathing difficulty, can be the initial potential life-threatening symptom of respiratory failure, also emphasised by the Airway, Breathing, Circulation (ABC)–principle in emergency care [3,4]. However, breathing difficulty encompasses a variety of clinical conditions, often, but not always due to respiratory diseases. Previous studies have estimated that dyspnoea and difficulty in breathing led to 5.8% - 7.3% of all dispatched ambulances, and was the fourth most frequent cause for the most urgent EMS responses [5,6]. In a Danish study, patients with dyspnoea as cause for dispatching an ambulance were found to have the second highest cumulative mortality rates (1-day: 4.6% and 30-day: 12.3%) among the EMS patients, only surpassed by the symptom of unconsciousness/cardiac arrest [7].

Diagnoses for EMS patients with acute dyspnoea are important to ensure the right treatment. Heart diseases and respiratory diseases have been found to be the most common diagnoses [8,9]. Few studies have reported outcome measurements, but a recent Danish study found that while 30-day mortality rate among EMS patients diagnosed with cardiovascular diseases, decreased from 20.1% in 2007 to 12.2% in 2014, the mortality rate was unchanged and substantial, around 12.5%, during the same period for EMS patients with respiratory diseases.

From the perspective of emergency departments contacts, a recent study found that respiratory diseases were the fifth most common diagnosis given, with the third highest 30-day mortality rate of 8.44%. Furthermore, a peak in the number of patients with respiratory diseases was observed in the very young children, and in the elderly. [10] Another study showed asthma stood out among young, and chronic obstructive pulmonary disease among the elderly patients admitted to an emergency department. [11]

The frequency, mortality, and underrated problem related to breathing difficulty, makes it crucial to study the prehospital patients with breathing difficulties further. Therefore, our aim was twofold, namely

1 to investigate the hospital diagnoses patterns and mortality rates of patients to whom EMS was dispatched due to breathing difficulty.

and

2 to investigate the initial main symptoms at the emergency call and mortality rates for EMS patients diagnosed with respiratory diseases in hospital.

## Materials and methods

### Ethics

The study was approved by the Danish Data Protection Agency (North Denmark Region record number 2008-58-0028 and project ID number 2016–80). Likewise, The Danish Patient Safety Authority approved the study (3-3013-1675/1) and gave permission to access prehospital patient medical records.

### Study design and setting

We performed a population-based historic cohort study on EMS patients to whom emergency ambulances were dispatched following a 1-1-2 call from January 2012 –September 2015.

To aid the interpretation of this study, a brief overview of the Danish prehospital system follows. As a tax supported system, the Danish health care is equally accessible for all citizens, including the prehospital system. The Danish emergency number (1-1-2) calls are answered by the Police, and since 2011, in case of a medical emergency, the call is forwarded to an Emergency Medical Coordination Centre. Here, healthcare professionals assess the severity and need for an ambulance by using a criteria based dispatch guideline, the Danish Index for Emergency Care, [12]. This is divided into 37 criteria corresponding to clinical signs, symptoms or incidents. As such, the healthcare professionals assess what they find to be the main issue over the phone, e.g. dyspnoea or *breathing difficulty* which is criteria number 28. The ambulance personnel do not assign a Danish Index for Emergency Care criteria. Below, we refer to the Danish Index for Emergency Care criteria as symptoms.

Every Danish citizen has a unique civil registration number, which enables linkage between registries and data. The regional Patient Administrative Systems contains data on patients’ diagnoses, health issues, and other reasons for contact to health services. The data is listed according to International Classification of Diseases, 10th edition (ICD-10). In Denmark, ICD-10 has been implemented since 1994, and it is required that any patient admitted to a hospital receive a diagnosis within the ICD-10 classifications [13,14].

The study took place in the North Denmark Region, which has approximately 587 000 inhabitants, corresponding to 10% of Denmark’s population, living in a combination of primarily rural and urban settings.

### Selection of participants

Of North Denmark Region citizens to whom an ambulance was dispatched following an 1-1-2 call, and subsequently brought to a hospital in the period January 2012 –September 2015 (45 months), we included two groups of patients:

1. EMS patients with *breathing difficulty* as the main symptom when calling 1-1-2.
2. EMS patients brought to a hospital by an ambulance after calling 1-1-2, who subsequently received a primary diagnosis within the ICD-10 main chapter X, Diseases of the respiratory system *(Respiratory diseases)*.

If a patient was transported by an emergency ambulance more than once, we only used the patient’s first contact in the study period. Patients without a known civil registration number and inter-hospital transportations were not included.

### Measurements

The logistic ambulance dispatch system, EVA 2000, provided technical data on dispatched emergency ambulances, symptom when calling 1-1-2, and patient identity (civil registration number). In cases where healthcare professionals did not assign a symptom at the 1-1-2 call, we noted the symptom as *not registered*.

We used the patients’ first primary diagnosis given in hospital according to ICD-10, which was retrieved from the regional Patient Administrative System. If a patient was given a non-specific primary diagnosis (ICD-10 main chapters XVIII, Symptoms, signs and abnormal clinical and laboratory findings, not elsewhere classified *(symptoms and signs)* and XXI, Factors influencing health status and contact with health services *(other factors*)), we searched for a more specific diagnosis during the hospital stay. Data on vital status, i.e. date of death, was retrieved from the Danish Civil Registration System.

### Outcomes

The main outcome was 1- and 30-day mortality rates. Patient age, sex, symptom when calling 1-1-2, and primary diagnosis given in hospital, were described.

The study’s two groups; patients with the symptom *breathing difficulty* and patients diagnosed with *respiratory diseases*, will be described separately.

### Analysis

Data were anonymised for statistical analysis. The results are presented as descriptive statistics with measures of frequency for the distribution of ICD-10 diagnoses and symptoms.

We used the Kaplan-Meier estimator to calculate 1-day and 30-day mortality. Patients who received a diagnosis unmistakably related to the certain death of the patient, were not included (the ICD-10 diagnoses “sudden cardiac death so described”, “other ill-defined and unspecified causes of mortality”, and the specific Danish code “cardiac death according to the Danish Health Act §176”). The mortality rates are presented as percentages with 95% confidence intervals and cumulative number of deaths. Only symptoms and ICD-10 main chapters with more than 100 patients are presented.

Stata/MP 15.1 (StataCorp LLC, Texas, USA) was used for all statistical analyses.

## Results

### Characteristics of study subjects

**Fig 1:**
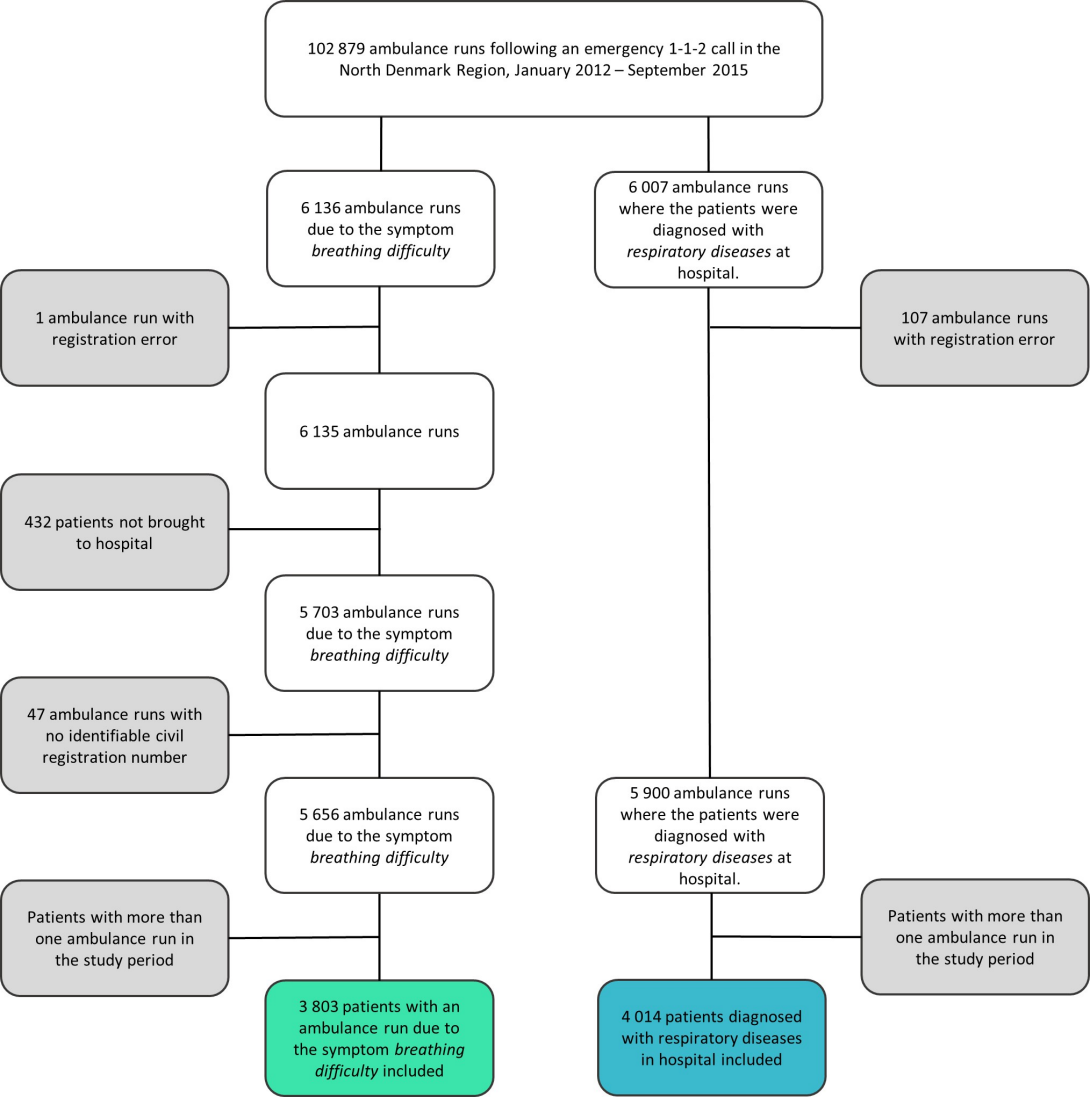
Flowchart for included ambulance runs. The included (white boxes) and excluded (grey boxes) ambulance runs and corresponding number of patients in the study period).

**Fig 2:**
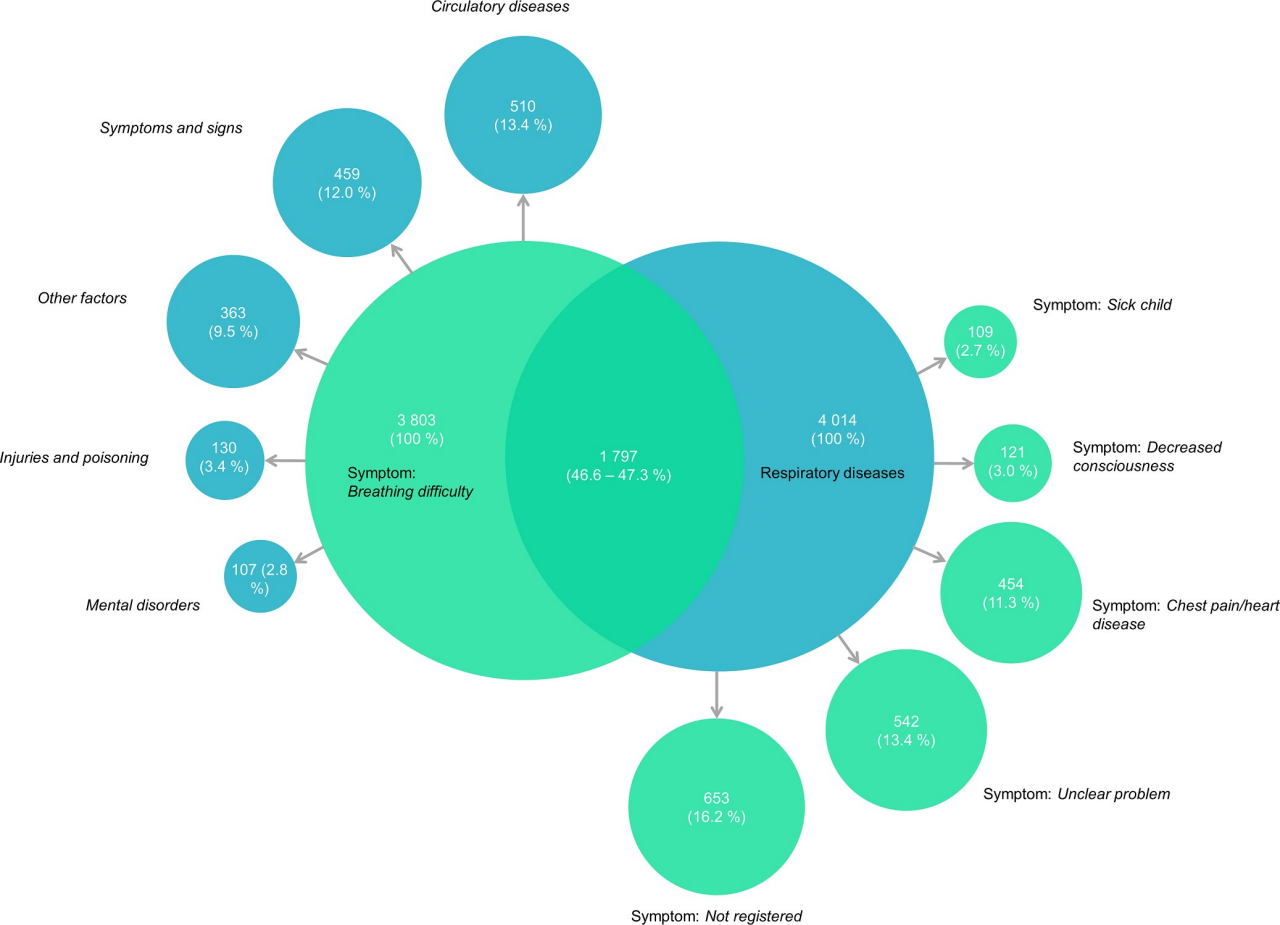
Symptoms and diagnoses overview. Diagram showing the relation between symptoms when calling the emergency number 1-1-2 (green circles) and primary diagnoses given in hospital, following a 1-1-2 call and dispatched ambulance (blue circles). Shows number of patients and percentage of corresponding group. Circle sizes are relative to number of patients.

In the study period a total of 102 879 emergency ambulances were dispatched (Fig 1). In total 6 136 were dispatched with *breathing difficulty* as the main symptom. Simultaneously, amongst the total ambulance runs, 6 007 resulted in a hospital contact with *respiratory diseases* as primary diagnosis. After exclusion of missing values, errors and multiple runs (Fig 1.), we included a total of 3 803 EMS patients who called 1-1-2 due to the symptom *breathing problem* and 4 014 EMS patients who were diagnosed with *respiratory diseases* in hospital. There was an overlap of 1 797 EMS patients between the two groups. This overlap is displayed in Fig 2.

The following results are divided separately into the study’s two groups.

### Main results

#### EMS patients with breathing difficulty as the main symptom when calling 1-1-2

A total of 3 803 individual patients were identified. Their median age was 69 (Interquartile range 53–79) and 50.0% of them were women.

Nearly half, 47.3% received a primary diagnosis within *respiratory diseases* in hospital. ICD-10 main chapter IX, Diseases of the circulatory system (c*irculatory diseases)* constituted 13.4% followed by the non-specific diagnoses: *symptoms and signs* and *other factors* at 12% and 9.6% respectively (Table 1).

**Table 1:** Primary diagnoses in hospital for patients with symptom breathing difficulty.

Diagnoses	N	Percent
***Respiratory diseases***	**1 797**	**47.25**
*J441*: *Chronic obstructive pulmonary disease with acute exacerbation*, *unspecified*	459	25.54
*J189*: *Pneumonia*, *unspecified*	363	20.20
*J449*: *Chronic obstructive pulmonary disease*, *unspecified*	185	10.29
*J960*: *Acute respiratory failure*	132	7.35
*J459*: *Asthma*, *unspecified*	110	6.12
***Circulatory diseases***	**510**	**13.41**
*I509*: *Heart failure*, *unspecified*	57	11.18
*I489*: *Atrial fibrillation or atrial flutter*, *unspecified*	53	10.39
*I214*: *Non-STEMI*	36	7.06
*I219*: *Acute myocardial infarction*, *unspecified*	31	6.08
*I269A*: *Pulmonary embolism*, *unspecified*	30	5.88
***Symptoms and signs***	**459**	**12.07**
*R060*: *Dyspnoea*	143	31.15
*R064*: *Hyperventilation*	91	19.83
*R074*: *Chest pain*, *unspecified*	33	7.19
*R539F*: *Malaise*	25	5.45
*R559*: *Syncope or collapse*	18	3.92
***Other factors***	**363**	**9.55**
*Z039*: *Observation for suspected disease or condition*, *unspecified*	229	63.09
*Z038*: *Observation for other suspected diseases and conditions*	36	9.92
*Z768*: *Persons encountering health services in other specified circumstances*	21	5.79
*Z035*: *Observation for other suspected cardiovascular diseases*	19	5.23
*Z03*: *Medical observation and evaluation for suspected diseases and conditions*	11	3.03
***Injuries and poisoning***	**130**	**3.42**
*S202*: *Contusion of thorax*	18	13.85
*S223*: *Fracture of rib*	11	8.46
*T783*: *Angioneurotic oedema*	5	3.85
*S060*: *Concussion*	4	3.08
*T784*: *Allergy*, *unspecified*	4	3.08
***Mental disorders***	**107**	**2.81**
*F100*: *Mental and behavioural disorders due to use of alcohol* : *acute intoxication*	23	21.50
*F419*: *Anxiety disorder*, *unspecified*	18	16.82
*F410*: *Panic disorder [episodic paroxysmal anxiety]*	10	9.35
*F102*: *Mental and behavioural disorders due to use of alcohol* : *dependence syndrome*	7	6.54
*F101*: *Mental and behavioural disorders due to use of alcohol* : *harmful use*	5	4.67
***Remaining***	**437**	**11.49**
**Total**	**3 803**	**100**

The most frequent primary diagnoses given in hospital according to ICD-10. Includes 3 803 patients who had the symptom breathing difficulty at the emergency 1-1-2 call and an ambulance dispatched. The five most frequent specific diagnoses are included for each main ICD-10 chapter, with percentage of their respective main chapter. ICD-10: International Classification of Diseases, 10th edition.

**Fig 3:**
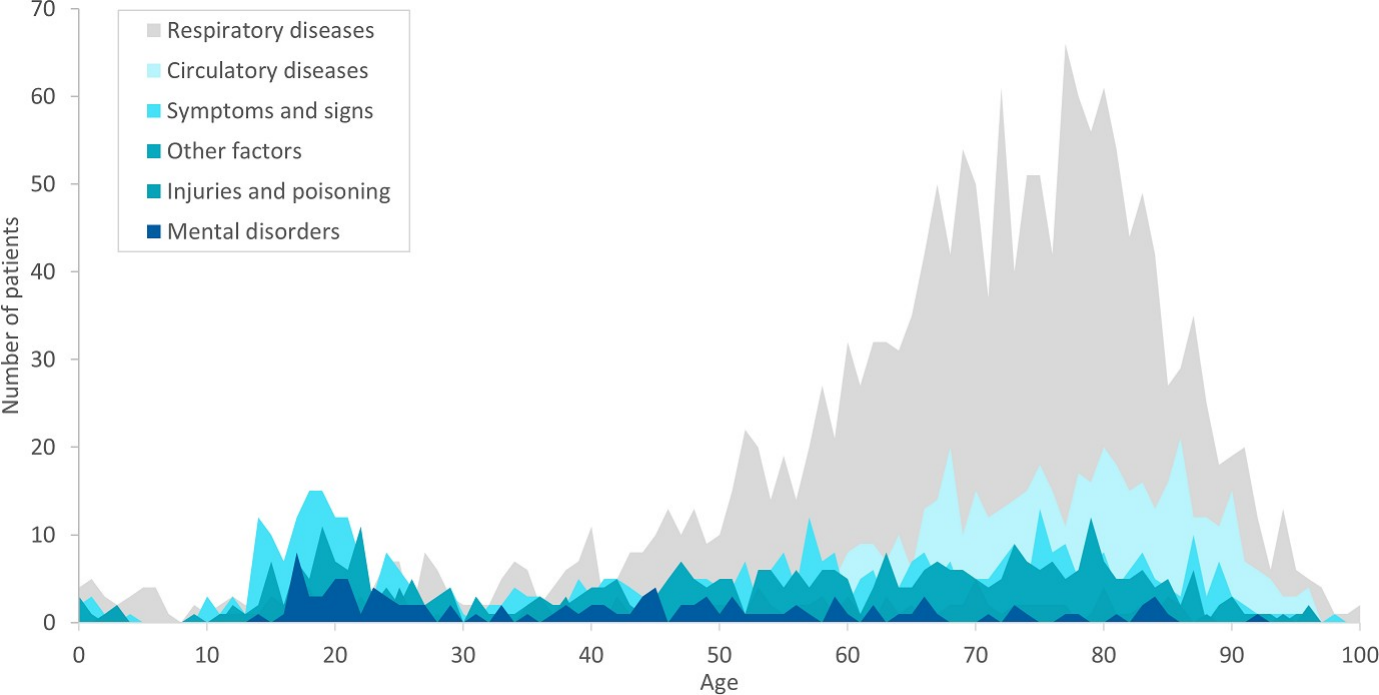
Primary diagnoses in hospital and age. Graph of individual primary diagnoses given in hospital according to ICD-10 main chapters. The graph includes 3 803 patients to whom an emergency ambulance was dispatched due the symptom breathing difficulty. ICD-10: International Classification of Diseases, 10th edition.

ICD-10 main chapter XIX, Injury, poisoning and certain other consequences of external causes (*injuries and poisoning*) and main chapter V, Mental and behavioural disorders (*mental disorders*) were given to 3.4% and 2.8% of the patients with the symptom *breathing difficulty*, most often in young adults, while *respiratory* and *circulatory diseases* increased with age (Fig 3). When looking closer at the diagnoses of *symptoms and signs*, half of the subcategories were related to breathing difficulties, without clear ethology, i.e. R06.0 Dyspnoea and R06.4 Hyperventilation. The highest 1-day and 30-day mortality rates were found within *circulatory diseases* (7.9% and 17.7% respectively), followed by *respiratory diseases* and *other factors* as seen in Table 2. Total number of deaths were highest among *respiratory diseases* with 232 patients, followed by *circulatory diseases* and both *symptoms and signs* and *other factors*. Of all deaths at day 30, only few patients, 0.8% (30 patients), were diagnosed with acute myocardial infarction or cardiac arrest.

**Table 2:** Mortality according to diagnoses.

Diagnoses	1-day mortality rate (percent, CI)	Cumulative number of deaths Day 1	30-day mortality rate (percent, CI)	Cumulative number of deaths Day 30	
*Total*	3.40 (2.88 to 4.00)	137	13.21 (12.20 to 14.30)	531
*Respiratory diseases*	3.62 (2.85 to 4.59)	65	12.95 (11.48 to 14.60)	232
*Circulatory diseases*	7.91 (5.86 to 10.62)	40	17.65 (14.59 to 21.27)	89
*Symptoms and signs*	0.88 (0.33 to 2.33)	4	5.77 (3.97 to 8.36)	26
*Other factors*	3.04 (1.69 to 5.42)	11	9.17 (6.60 to 12.65)	33
*Injuries and poisoning*	2.31 (0.75 to 6.98)	3	3.86 (1.62 to 9.02)	5
*Mental disorders*	0.00 (0.00 to 0.00)	0	3.74 (1.42 to 9.65)	4

1- and 30-day mortality for 3 803 patients who had the symptom breathing difficulty at the 1-1-2 call and an ambulance dispatched. Separated by ICD-10 main chapters.

CI: 95% Confidence interval. ICD-10: International Classification of Diseases, 10th edition.

#### EMS patients diagnosed with respiratory diseases in hospital

**Fig 4:**
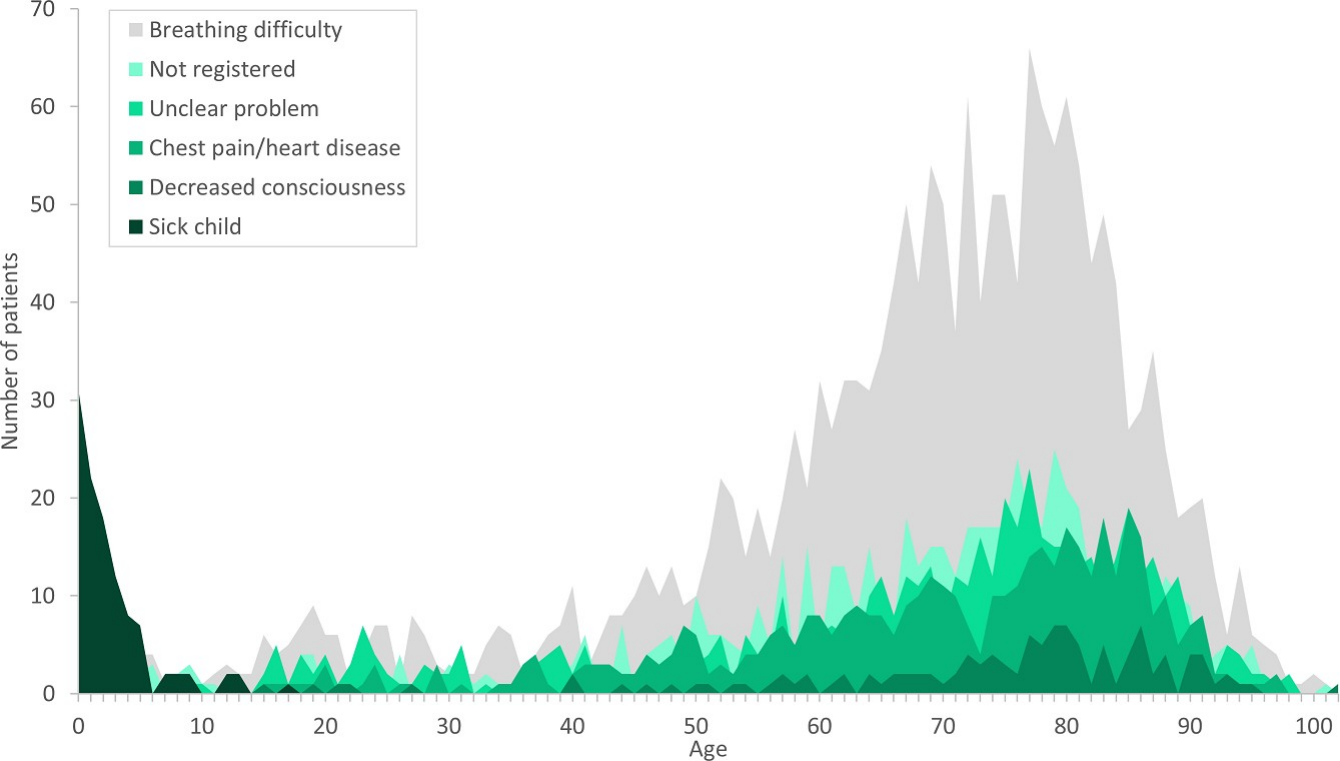
Symptom and age. Graph of individual symptoms when calling 1-1-2 according to age. The graph includes 4 014 patients diagnosed with respiratory diseases in hospital, following an emergency 1-1-2 call and dispatched ambulance.

A total of 4 014 individual patients were identified. The median age was 71 (Interquartile range 57–80) and 46% were women. The initial symptoms when calling 1-1-2 were for the majority *breathing difficulty*, 44.8%, *(*1 797 patients), followed by *unclear problem* with 13.4% and *chest pain* 11.3%. In patients diagnosed with respiratory diseases, 3.0% (121 patients) had the symptom *decreased consciousness* when calling 1-1-2. All symptoms were prominent among patients above 50 years old. Among young children diagnosed with respiratory diseases, the most frequent symptom (criteria assessed over the phone for dispatching the ambulance) was *sick child* (Fig 4). For 16.2% (653 patients) the symptom was *not registered*, thus we do not know the initial symptom at the emergency call.

Over-all 1- and 30-day mortality rates was 3.7% and 12.5%. The symptoms *decreased consciousness* had the highest mortality rates of 4.1% and 19.1%, followed by *breathing difficulties*, and *unclear problem* (see Table 3). Mortality rates were similar in patients with symptom *not registered*. Total number of deaths were found to be highest among the symptom *breathing difficulties* (232 patients), followed by *unclear problem*, and *chest pain/heart disease*.

**Table 3:** Mortality according to symptom.

Hierarchy	1-day mortality rate (percent, CI)	Cumulative number of deaths Day 1	30-day mortality rate (percent, CI)	Cumulative number of deaths Day 30
*Total*	3.72 (3.16 to 4.37)	148	12.52 (11.51 to 13.62)	480
*Breathing difficulty*	3.62 (2.85 to 4.59)	65	12.95 (11.48 to 14.60)	232
*Unclear problem*	2.59 (1.54 to 4.33)	14	13.14 (10.56 to 16.28)	71
*Chest pain/heart disease*	1.10 (0.46 to 2.63)	5	6.63 (4.68 to 9.35)	30
*Decreased consciousness*	4.13 (1.74 to 9.64)	5	19.10 (13.13 to 27.34)	23
*Not registered*	3.98 (2.73 to 5.79)	26	14.90 (12.38 to 17.88)	97

1- and 30-day mortality for 4 014 patients diagnosed with respiratory diseases in hospital, following an emergency 1-1-2 call and ambulance dispatch. Separated by symptom when calling 1-1-2.

CI– 95% Confidence interval.

## Discussion

We found the most frequent diagnoses given to EMS patients calling 1-1-2 with breathing difficulties, to be *respiratory diseases*, *circulatory diseases*, *symptoms and signs*, and *other factors*. 1- and 30-day mortality rates were over-all 3.4% and 13.2%. For EMS patients diagnosed with *respiratory diseases*, we found that the symptoms *breathing difficulty*, *unclear problem*, and *chest pain/heart disease* were the most frequent. Also, here, the 1- and 30-day mortality rates were similar, over-all 3.7% and 12.5%.

We chose to include both EMS and hospital data which have different coding processes. However, this aided the exhaustiveness and representativeness of the study.

For 16% of the patients there was no information on the criteria for dispatching the ambulance possibly because registrations were done manually by the call-takers. The *not registered* symptoms at the emergency call could have contained specific symptoms, resulting in a shift of the frequencies reported in this study. Likewise, it is possible that patients with other symptoms than *breathing difficulties* could have experienced dyspnoea, as it is present in other conditions. A greater level of detail may have been obtained if the patients’ medical records were accessed. However, hospital diagnoses are part of the daily clinical practice and registration of acute admissions in the Patient Administrative System have previously been found to have a high validity. [14,15] Furthermore, we used the criteria for dispatching an ambulance, assessed by healthcare professionals at the emergency call. This is the first available data, regarding the situation. More detailed clinical information may have been obtained if the patients’ prehospital medical record were accessed, providing information from ambulance personnel in direct contact with the patient, in contrast to the initial phone assessment.

Furthermore, the choice of using the patients’ first contact in the study period might have resulted in a lower mortality rate, than if the patients’ last contact had been used, due to possible repeated users and patients with chronic diseases. Thus, our results concerning mortality is not overestimated. Likewise, the exclusion of patients without a valid civil registration number and patients not brought to a hospital, could have shifted the mortality rates. However, we did not have information of the possible date of death for these patients.

Apart from respiratory diseases, we found that heart diseases are prominent among EMS patients presenting breathing difficulty. This is consistent with studies from the USA [8], Australia and New Zeeland [9], and Germany [16]. However, our study also revealed that non-specific diagnoses were frequently applied to patients with breathing difficulty. A high number of non-specific diagnoses have also been found in previous Danish studies. [10,17] This underlines the complexity of dyspnoea and stresses the need for further research of this patient group in EMS, to gain insight in the patient population that the EMS staff faces.

It is important to note that our study focused on the symptoms as presented at the initial contact, the call to the Emergency Medical Coordination Centre over the phone, which is important because this first assessment of the patient determines the EMS response and patient care pathway. This contrasts with the USA study, where the patients’ main symptoms were defined by the EMS personnel on scene. [8] In the Australian and New Zeeland study, it was defined by the emergency department personnel. [9] Finally, in the German study the treating physician specified the patient’s chief complaint after arrival to the Emergency Department. [16] The German study also included mortality as an outcome measure and found an in-hospital mortality of 9.4% for patients with dyspnoea as the chief complain. This mortality rate is similar to the 30-day mortality rate of 13% found in the current study.

We found that 13.2% of the patients with the initial symptom *breathing difficulty* were deceased within 30 days from the 1-1-2 call. This is consistent with a recent Danish study which found a 30-day mortality rate of 12.6% (CI: 11.9–13.3) for patients with *breathing difficulty* as the main symptom when calling 1-1-2 [7]. However, we also found that only less than one percent of these patients had diagnoses related to acute myocardial infarction or cardiac arrest. This emphasises the severity of *breathing difficulty* beyond only circulatory diseases.

Our study found *respiratory diseases* increased with age and were prominent among the elderly. This is supported by a study from a USA emergency department, which examined trends in emergency department-use by elderly adults. The study identified shortness of breath and chest pain as the two most common reasons for emergency department visits. [18] With a median age of 69 year for patients with the symptom *breathing difficulty*, and a median age of 71 years for patients diagnosed with *respiratory diseases* in hospital, the elderly represents the majority of patients in this study. The variation in diagnoses according to age groups, is interesting for future studies, due to knowledge needed in emergency departments or intensive care units.

In conclusion, the over-all 30-day mortality rates of 13.2% and 12.5% for the symptom and diagnosis respectively, alongside the distribution of symptoms and diagnoses, suggest the breathing difficulty patient group is complex and has severe health problems. Consequently, these findings may be able to raise awareness towards the patient group, and thereby increase focus on diagnostics and treatment to improve the patient outcome.
